# Supplementary material for: Scale-up costs and societal benefits of psychological interventions for alcohol use and depressive disorders in India
Source: PLOS Glob Public Health. 2023 Sep 14;3(9):e0002017. doi: 10.1371/journal.pgph.0002017 (PMC10501670; doi:10.1371/journal.pgph.0002017)
Supplement: Attachment — Submitted filename: Scale up cost-Review.docx [file pgph.0002017.s002.docx]

**ABSTRACT**

**The author discusses the necessity for CAP and HAP finance to be scaled up in India, as well as estimations of the total benefits of such financial scaling.**

Lines 10 to 19 of the abstract must be updated to provide a clear comprehension of the desired methodology and outcomes. For example, *‘Scale-up costs were calculated for meeting total or unmet needs Societal benefit estimates based on averted disease burden were calculated using human capital and value of life-year approaches’* doesn’t entirely make sense.

**INTRODUCTION**

1. Paragraph 1, line 3 asserts that India requires three psychiatrists per million, but I disagree. According to data from OECD countries and the USA, the ideal number is between 10-20 per 100,000. Additionally, the study cited in the paragraph estimates that there are only three psychiatrists per 100,000 in India. The paper also laments the scarcity of doctors, which makes it challenging to meet the current standards, which are generally inadequate. The sentence should be updated to reflect accurate facts.
2. The first line of the second paragraph is too long and difficult to understand. To improve clarity, authors should use shorter sentences and choose their words carefully. For instance:

Research has shown that primary care lay-counseling with a brief intervention called HAP is both effective and cost-effective, as compared to ABC.

3. In the same way, it is possible to rephrase line 7 in paragraph 2.
4. *“Scaling up programs such as CAP and HAP at the national level can mitigate the treatment gaps, relieve the workload pressure on the existing limited psychiatric workforce, and lead to better population health and broader societal outcomes.”*

I feel the above should be presented as monitored or governed, professionally trained lay-counselling services scaling up. See reference below.

Connolly, S. M., Vanchu-Orosco, M., Warner, J., Seidi, P. A., Edwards, J., Boath, E., & Irgens, A. C. (2021). Mental health interventions by lay counsellors: a systematic review and meta-analysis. Bulletin of the World Health Organization, 99(8), 572–582. https://doi.org/10.2471/BLT.20.269050

**MATERIALS AND METHODS**

**General comment**

Mathematical or diagrammatic summary of the calculations would prove essential to follow the arguments raised as far as the scale-up estimates are concerned. Similarly, the same applies to the economical benefits.

I didn’t follow how all the scale up estimates were done based on 2016 data as referenced in the sentence *“Population-level national values of self-reported treatment gaps for major depressive disorder (85.2%) and AUD (86.3%) were obtained from the National Mental Health Survey (2016) [15]”* in section 2.1.

Additionally, for the sentence “Values related to the value of life-year approach (see ahead) were based on previous studies [16]”, please provide a brief description and what values were used in your study.

I would find it interesting to demonstrate (as earlier mentioned) how each variable used was estimated and grounds that support the use of the variables in literature.
