# Supplementary material for: Scale-up costs and societal benefits of psychological interventions for alcohol use and depressive disorders in India
Source: PLOS Glob Public Health. 2023 Sep 14;3(9):e0002017. doi: 10.1371/journal.pgph.0002017 (PMC10501670; doi:10.1371/journal.pgph.0002017)
Supplement: Attachment — Submitted filename: 5_JUL_23_SZ_Response_Letter_MH_Scale_up.docx [file pgph.0002017.s003.docx]

Response Letter

Journal Requirements:

1. Please provide separate figure files in .tif or .eps format.

For more information about figure files please see our guidelines: LINK

https://journals.plos.org/globalpublichealth/s/figures
https://journals.plos.org/globalpublichealth/s/figures#loc-file-requirements

**Authors’ Response: The revised submission has all figure files in .tiff format.**

2. Please send a completed 'Competing Interests' statement, including any COIs declared by your co-authors. If you have no competing interests to declare, please state "The authors have declared that no competing interests exist". Otherwise please declare all competing interests beginning with the statement "I have read the journal's policy and the authors of this manuscript have the following competing interests:"

**Authors’ Response: A Competing Interests Statement has been added to the revised submission.**

Reviewer Comments:

Reviewer #1: The manuscript was clear, well written according to the PLOS Global public health's publication criteria. Health expenditure and country's GDP and the annual costs of scaling up CAP and HAP were estimated.

**Authors’ Response: We thank the reviewer for their comments.**

Reviewer #2: ABSTRACT
The author discusses the necessity for CAP and HAP finance to be scaled up in India, as well as estimations of the total benefits of such financial scaling.

Lines 10 to 19 of the abstract must be updated to provide a clear comprehension of the desired methodology and outcomes. For example, ‘Scale-up costs were calculated for meeting total or unmet needs, societal benefit estimates based on averted disease burden were calculated using human capital and the value of life-year approaches’ doesn’t entirely make sense.

**Authors’ Response: We thank the reviewer for this comment and understand and agree with their concern. There was a punctuation error that we have now corrected. Additionally, we have rephrased the part for clarity. Unfortunately, the journal word limit on the abstract does not let us explain more details of the methods/outcomes in the abstract. However, we hope that the changes and corrections made help alleviate the reviewer’s previous concerns.**

INTRODUCTION
1. Paragraph 1, line 3 asserts that India requires three psychiatrists per million, but I disagree. According to data from OECD countries and the USA, the ideal number is between 10-20 per 100,000. Additionally, the study cited in the paragraph estimates that there are only three psychiatrists per 100,000 in India. The paper also laments the scarcity of doctors, which makes it challenging to meet the current standards, which are generally inadequate. The sentence should be updated to reflect accurate facts.

**Authors’ Response: We apologize for the error and thank the reviewer for pointing this out. We have now updated the numbers based on the paper from eminent Indian psychiatrists at the Department of Psychiatry, National Institute of Mental Health and Neurosciences who were involved in conducting the National Mental Health Survey.**

2. The first line of the second paragraph is too long and difficult to understand. To improve clarity, authors should use shorter sentences and choose their words carefully. For instance:
Research has shown that primary care lay-counseling with a brief intervention called HAP is both effective and cost-effective, as compared to ABC.

**Authors’ Response: We thank the reviewer for this comment. We have now edited the sentence into two for clarity in the received manuscript.**

3. In the same way, it is possible to rephrase line 7 in paragraph 2.

**Authors’ Response: We thank the reviewer for this comment. We have now edited the sentence into two for clarity in the received manuscript.**

4. “Scaling up programs such as CAP and HAP at the national level can mitigate the treatment gaps, relieve the workload pressure on the existing limited psychiatric workforce, and lead to better population health and broader societal outcomes.”

I feel the above should be presented as monitored or governed, professionally trained lay-counselling services scaling up. See reference below.

Connolly, S. M., Vanchu-Orosco, M., Warner, J., Seidi, P. A., Edwards, J., Boath, E., & Irgens, A. C. (2021). Mental health interventions by lay counselors: a systematic review and meta-analysis. Bulletin of the World Health Organization, 99(8), 572–582. <https://doi.org/10.2471/BLT.20.269050>

**Authors’ Response: We thank the reviewer for the comment. We have now added a statement to acknowledge this along with the citation.**

MATERIALS AND METHODS
General comment
Mathematics or diagrammatic summary of the calculations would prove essential to follow the arguments raised as far as the scale-up estimates are concerned. Similarly, the same applies to the economic benefits.

**Authors’ Response: We thank the reviewer for this comment. We have now added a flow chart describing all the calculations. See Figure 1 in the revised manuscript.**

I didn’t follow how all the scale up estimates were done based on 2016 data as referenced in the sentence “Population-level national values of self-reported treatment gaps for major depressive disorder (85.2%) and AUD (86.3%) were obtained from the National Mental Health Survey (2016) [15]” in section 2.1.

**Authors’ Response: The treatment gap values were taken from the 2016 survey. This was the only available and most recent national-level survey reporting mental healthcare treatment gaps. Rest all the data including prevalence, DALY, GDP per capita, etc. are for 2019. We have now also added this in the limitations under Discussion. See, the seventh point there.**

Additionally, for the sentence “Values related to the value of life-year approach (see ahead) were based on previous studies [16]”, please provide a brief description and what values were used in your study.

**Authors’ Response: The values have been described in section 2.3. We have mentioned this more clearly in the received manuscript now.**

I would find it interesting to demonstrate (as earlier mentioned) how each variable used was estimated and grounds that support the use of the variables in literature.

**Authors’ Response: We thank the reviewer for this comment. We described this in detail in the methods. Additionally, a flowchart of the analytical plan has now been added to the revised manuscript that depicts variables and calculations. See Figure 1 in the revised manuscript.**
